# Oxygen Consumption with High-Flow Nasal Oxygen versus Mechanical Ventilation— An International Multicenter Observational Study in COVID–19 Patients (PROXY–COVID)

**DOI:** 10.4269/ajtmh.22-0793

**Published:** 2023-03-27

**Authors:** Michela Botta, Oriol Caritg, David M.P. van Meenen, Andrés Pacheco, Anissa M. Tsonas, Willemijn E. Mooij, Alessia Burgener, Tosca Manrique Hehl, Gentle S. Shrestha, Janneke Horn, Pieter R. Tuinman, Frederique Paulus, Oriol Roca, Marcus J. Schultz

**Affiliations:** 1 Department of Intensive Care, Amsterdam University Medical Centers, location AMC, Amsterdam, The Netherlands;; 2 Department of Intensive Care, Vall d’Hebron University Hospital, Barcelona, Spain;; 3 Department of Critical Care Medicine, Tribhuvan University Teaching Hospital, Kathmandu, Nepal;; 4 Amsterdam Neurosciences, Amsterdam UMC Research Institute, Amsterdam, The Netherlands;; 5 Department of Intensive Care, Amsterdam University Medical Centers, location VUMC, Amsterdam, The Netherlands;; 6 Urban Vitality, Centre of Expertise, Faculty of Health, Amsterdam University of Applied Sciences, Amsterdam, The Netherlands;; 7 Department of Intensive Care, Parc Taulí de Sabadell University Hospital, Barcelona, Spain;; 8 Departament de Medicina, Universitat Autònoma de Barcelona, Bellaterra, Spain;; 9 Ciber Enfermedades Respiratorias, Instituto de Salud Carlos III, Madrid, Spain;; 10 Mahidol–Oxford Tropical Medicine Research Unit, Mahidol University, Bangkok, Thailand;; 11 Nuffield Department of Medicine, University of Oxford, Oxford, United Kingdom

## Abstract

The COVID–19 pandemic led to local oxygen shortages worldwide. To gain a better understanding of oxygen consumption with different respiratory supportive therapies, we conducted an international multicenter observational study to determine the precise amount of oxygen consumption with high-flow nasal oxygen (HFNO) and with mechanical ventilation. A retrospective observational study was conducted in three intensive care units (ICUs) in the Netherlands and Spain. Patients were classified as HFNO patients or ventilated patients, according to the mode of oxygen supplementation with which a patient started. The primary endpoint was actual oxygen consumption; secondary endpoints were hourly and total oxygen consumption during the first two full calendar days. Of 275 patients, 147 started with HFNO and 128 with mechanical ventilation. Actual oxygen use was 4.9-fold higher in patients who started with HFNO than in patients who started with ventilation (median 14.2 [8.4–18.4] versus 2.9 [1.8–4.1] L/minute; mean difference = 11.3 [95% CI 11.0–11.6] L/minute; *P* < 0.01). Hourly and total oxygen consumption were 4.8-fold (*P* < 0.01) and 4.8-fold (*P* < 0.01) higher. Actual oxygen consumption, hourly oxygen consumption, and total oxygen consumption are substantially higher in patients that start with HFNO compared with patients that start with mechanical ventilation. This information may help hospitals and ICUs predicting oxygen needs during high-demand periods and could guide decisions regarding the source of distribution of medical oxygen.

## INTRODUCTION

The coronavirus disease 2019 (COVID–19) pandemic taught us that oxygen scarcity is a serious but, above all, realistic scenario in healthcare systems worldwide.^1^^–^^3^ Indeed, in several parts of the world, there have been shorter or longer periods of oxygen shortages, both in high-income and in low- and middle-income countries.^4^^–^^7^ Shortages may have been exacerbated by the increased use of high-flow oxygen supplementation, often called “high-flow nasal oxygen” (HFNO).^8^^–^^10^

Although it is obvious that HFNO consumes more oxygen than supplementation with lower flow oxygen support such as noninvasive and invasive ventilation, it remains unclear precisely how great the difference in oxygen consumption is. A difference in oxygen consumption at a patient level may be less important, but at a department or hospital level, it is vital to understand this difference for various reasons. For instance, it could help intensive care unit (ICU) staff predict department oxygen needs during high-demand periods. Furthermore, it could help hospitals in decisions regarding the source of medical oxygen (e.g., liquid oxygen or pressure swing adsorption plant, oxygen concentrators or oxygen cylinders) as well as the infrastructure (e.g., oxygen concentrators, oxygen cylinders, or piped oxygen).^11^^–^^13^

To improve our understanding of oxygen consumption with HFNO versus mechanical ventilation, we conducted a study, “Oxygen Consumption with High-Flow Nasal Oxygen versus Mechanical Ventilation—An International Multicenter Observational Study in COVID–19 patients” (PROXY–COVID). PROXY-COVID was a substudy of a larger observational study in ICU patients with acute hypoxemic respiratory failure due to COVID–19. Our aim was to compare the amount of supplemental oxygen used with HFNO versus mechanical ventilation during the first two full calendar days of oxygen support in the ICU.

## MATERIALS AND METHODS

### Study design.

PROXY-COVID is a substudy of a large retrospective observational study, “Practice of Adjunctive Treatments in Critically Ill COVID–19 Patients” (PRoAcT-COVID). PRoAcT-COVID is a study that focused on care processes, including respiratory support, in critically ill COVID–19 patients in the first year of the outbreak in Europe. PROXY-COVID was performed in the Academic Medical Center and the Free University Medical Center, two academic hospitals in Amsterdam, The Netherlands, and in the Vall d’Hebron University Hospital, an academic hospital in Barcelona, Spain, from October 2020 to December 2020. The Institutional Review Boards of the participating hospitals approved the study protocol of PRoAcT-COVID and this substudy and waived the need for individual patient informed consent because of the observational nature of the investigation (11 December 2020; W20_526 # 20.583). PRoAcT-COVID and the substudy PROXY-COVID are registered at clinicaltrials.gov (study identifier NCT04719182).

### Inclusion and exclusion criteria.

Patients were eligible for participation in this substudy if they were 1) aged > 18 years and 2) admitted to the ICU of one of the participating hospitals for 3) acute hypoxemic respiratory failure due to reverse transcriptase polymerase chain reaction–confirmed COVID–19. Patients were excluded if a patient did neither receive HFNO or mechanical ventilation during the first two full calendar days of ICU admission. We also excluded patients who received extracorporeal life support, including extracorporeal membrane oxygenation or extracorporeal CO_2_ removal within that timeframe.

### Data collected.

Demographics, comorbidities, home medication, and outcomes were collected at baseline. The Simplified Acute Physiology Score II was calculated using data collected in the first 24 hours after arrival in the ICU. Outcomes collected in this study were the last day of ventilation, last day in the ICU, last day in the hospital, and life status up to day 90.

For every 2 hours in the first two full calendar days, we collected the following data to determine oxygen consumption: oxygen supplementation mode in use (i.e., HFNO, noninvasive ventilation, or invasive ventilation). With HFNO, we collected the fraction of inspired oxygen (FiO_2_) and the airflow. With noninvasive ventilation and invasive ventilation, we collected FiO_2_ and the level of positive end-expiratory pressure, as well as the inspiratory tidal volume (V_T_) and the respiratory rate (RR). In addition, we collected the saturation of arterial oxygen by pulse oximetry (SpO_2_), and partial pressure of arterial oxygen, for every arterial blood gas analysis performed in the first two full days.

### Patient classification.

We created two groups of patients. Patients who started with HFNO were placed in the HFNO patient group; patients who started with mechanical ventilation, whether noninvasive or invasive, were placed in the ventilated patient group. In both groups, patients could have received the alternative means of oxygen supplementation (i.e., a patient that started with HFNO could continue with mechanical ventilation, and vice versa). In all patients, noninvasive ventilation and invasive ventilation were applied using high-end ICU ventilators.

### Calculations.

First, we calculated the amount of supplemental oxygen per minute at each timepoint. During HFNO, we used the following equation:

$$\text{Supplemental}\;\text{oxygen}\;\left(\text{L}/\text{minute}\right)=\text{air}\;\text{flow}\;\left(\text{L}/\text{minute}\right)*\left({\text{FiO}}_{2}-0.21\right)$$ [Eq. 1]

For patients under invasive ventilation or noninvasive ventilation, we used the following equation:

$$\text{Supplemental}\;\text{oxygen}\;\left(\text{L}/\text{minute}\right)={\text{V}}_{\text{T}}\left(\text{L}\right)*\text{RR}*\left({\text{FiO}}_{2}-0.21\right)$$ [Eq. 2]

Supplemental oxygen per minute was then calculated using Eqs. (1) and (2) at every timepoint (i.e., every 2 hours). The amount of supplemental oxygen used between each timepoint was calculated based on the assumption that the supplemental oxygen per minute did not change over the subsequent 2 hours. The total amount of supplemental oxygen used over the first full calendar day (Day 1) and the second full calendar day (Day 2) was calculated by adding the supplemental oxygen used between each timepoint. The supplemental oxygen per minute for every patient thus corresponds to the mean of all observations within the first two full days for each patient.

### Outcomes.

The primary outcome was oxygen consumption per minute (actual oxygen consumption) during the first two full calendar days of ICU admission. Secondary outcomes were oxygen consumption per hour (hourly oxygen consumption) and total oxygen consumption (total oxygen consumption) in the same time frame.

### Power calculation.

No formal sample size calculation was performed. The number of available patients served as the sample size.

### Statistical analysis plan.

Continuous data are reported as medians with interquartile ranges and categorical data as numbers with percentages.

The number of patients in the HFNO patients group and in the ventilated patients group are reported in the Consolidated Standards of Reporting Trials diagram. The groups were compared using a Wilcoxon–Mann–Whitney test for continuous data and a Fisher exact test for categorical data.

Missing values, when not present in more than 5% of the observations, were treated as follows: the mean between the two proximal observations was calculated for every missing variable (i.e., V_T_, RR, FiO_2_, airflow) to be able to calculate the supplemental oxygen as in Eqs. (1) and (2).

Actual oxygen consumption, hourly oxygen consumption, and total oxygen consumption at patient level were compared between the two groups using the Wilcoxon–Mann–Whitney test and presented in distribution graphs. Actual oxygen consumption was plotted over time in a line graph showing mean and standard deviation, in combination with FiO_2_ and with air flow during HFNO, and minute ventilation during ventilation.

Actual oxygen consumption was compared between the two groups and over time with repeated measures analysis of variance. Data were log transformed when skewed, and if significant, a pairwise comparison between the two groups for every time points was performed with Bonferroni correction.

In a preplanned subgroup analysis, patients that alternately received HFNO and ventilation in the first two full days were excluded so that only patients who exclusively received HFNO or ventilation could be compared.

All analyses were conducted in R Studio v. 4.0.3 (R Foundation, Vienna, Austria), and the significance level was set at 0.05.

## RESULTS

**Figure 1.**
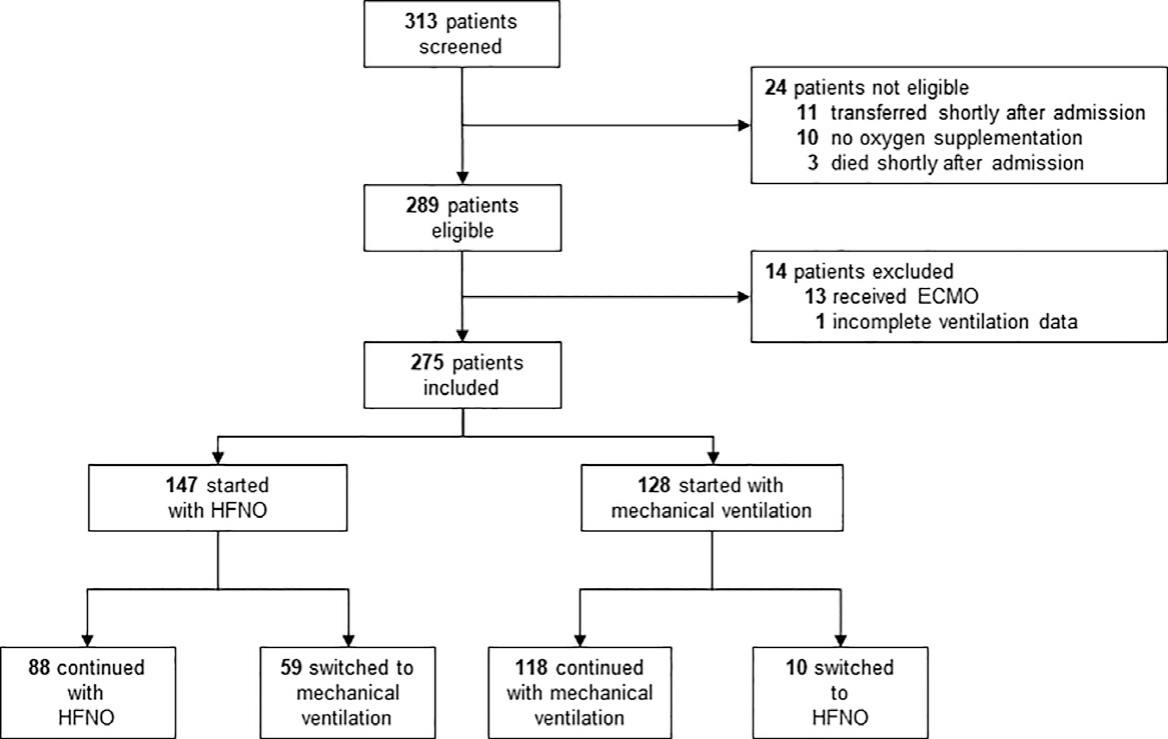
Consolidated Standards of Reporting Trials flow diagram. ECMO = extracorporeal membrane oxygenation; HFNO = high flow nasal oxygen.

Between October 1, 2020, and December 31, 2020, of a total of 313 screened patients, 289 were eligible for participation (Figure 1). Of these patients, 13 were excluded because they had received extracorporeal membrane oxygenation, and one was excluded for incomplete ventilation data. Of the remaining patients, 147 started with HFNO, and 128 started with mechanical ventilation. Patients were mostly male with a medical history of hypertension and diabetes mellitus (Table 1). Patients who started with HFNO were younger, less sick, and died less often than patients who started with ventilation.

**Table 1:** Baseline patient characteristics

Characteristic	HFNO patients (*N* = 147)	Ventilated patients (*N* = 128)	*P*
Age, years	61 (53–70)	65 (58–72)	0.02
Male gender, *n* (%)	101 (70.1)	93 (72.7)	0.69
Body mass index, kg/m^2^	28.7 (26.0–31.7)	28.7 (25.0–32.5)	0.63
SAPS II,* *n*/*N*	(129/147), 33 (27–39)	(102/128) 51 (41–63)	< 0.01
Comorbidities, *n* (%)			
Arterial hypertension	56 (38.1)	57 (44.5)	0.33
Cardiovascular disease	26 (17.7)	29 (22.7)	0.36
Diabetes mellitus	45 (30.6)	34 (26.6)	0.51
Chronic kidney disease	19 (12.9)	13 (10.2)	0.57
Pulmonary disease	17 (11.6)	20 (15.6)	0.38
Malignancy	13 (8.8)	11 (8.6)	1.00
Home medication, *n* (%)			
Systemic corticosteroids	12 (8.2)	7 (5.5)	0.48
ACE inhibitors	25 (17.1)	27 (21.1)	0.44
Angiotensin II receptor blockers	22 (15.1)	15 (11.7)	0.48
Beta blockers	26 (17.8)	27 (21.1)	0.54
Insulin	18 (12.3)	19 (14.8)	0.60
Oral antidiabetics	35 (24.0)	23 (18.0)	0.24
Statins	47 (32.2)	39 (30.5)	0.80
Calcium channel blockers	25 (17.1)	24 (18.8)	0.75
Anticoagulation	16 (11.0)	17 (13.3)	0.58
ICU mortality, *n* (%)	25 (17.0)	52 (40.9)	< 0.01

ACE = angiotensin converting enzyme; HFNO = high flow nasal oxygen; ICU = intensive care unit; SAPS = Simplified Acute Physiology Score. Data are median (plus interquartile range) or *n* (%).

* SAPS II was not available for all patients.

### Mode of oxygen supplementation.

**Figure 2.**
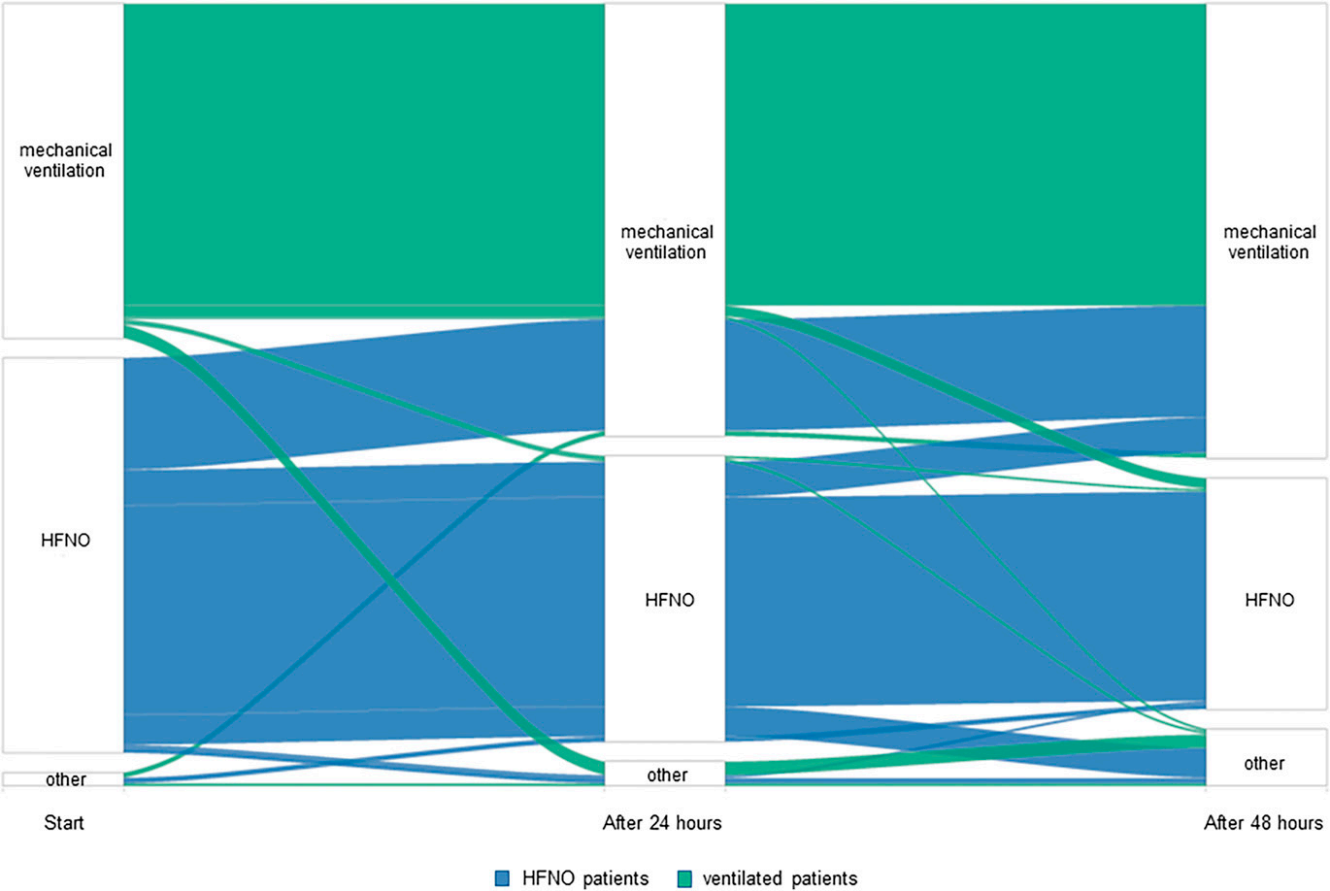
Mode of oxygen supplementation over time. HFNO = high flow nasal oxygen.

Of the HFNO patients, 54% continued with this mode of oxygen supplementation for the complete first two full days; the other patients switched to ventilation (37%) or low-flow oxygen (9%) within this timeframe. Of the ventilated patients, the vast majority continued with this mode: 10% switched to HFNO or another mode of oxygen supplementation (Figure 2 and Supplemental Figure 1).

### Oxygen consumption.

**Figure 3.**
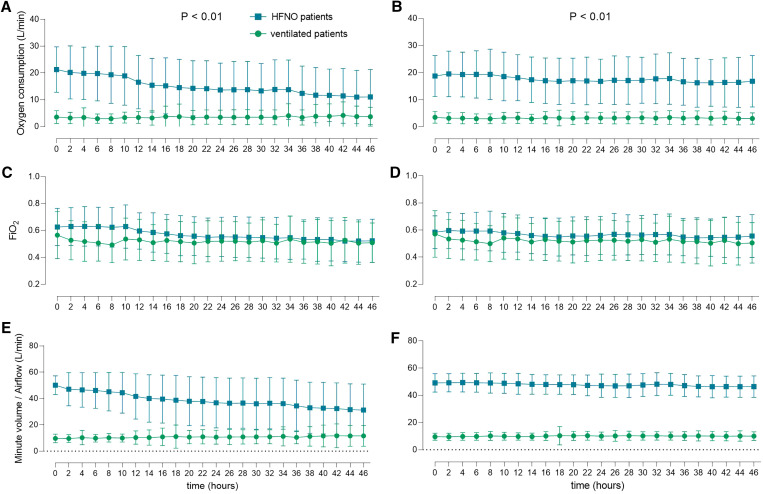
Actual oxygen consumption, fraction of inspired oxygen (FiO_2_) and airflow/minute volume over time. Panels on the left show the primary analysis, panels on the right show the preplanned subgroup analysis. *P* values refer to the repeated measures analysis of variance. HFNO = high flow nasal oxygen.

**Figure 4.**
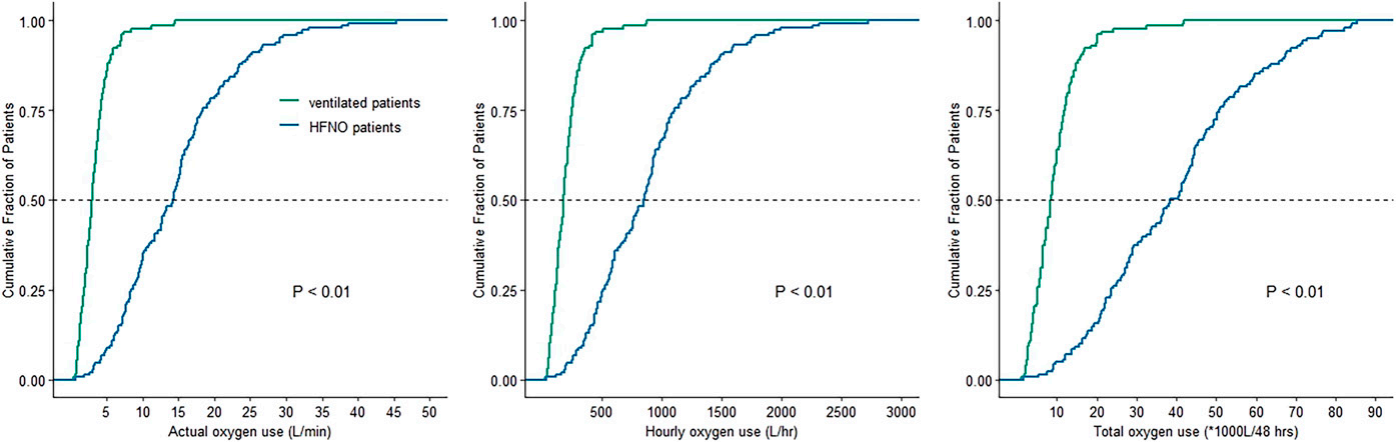
Cumulative frequency distribution of actual oxygen use, hourly oxygen use, and total oxygen use.* P* values refer to the Wilcoxon–Mann–Whitney test. HFNO = high flow nasal oxygen.

Actual oxygen consumption was 4.9-fold higher in HFNO patients compared with ventilated patients (median 14.2 [8.4–18.4] versus 2.9 [1.8–4.1] L/minute; mean difference = 11.3 [95% CI: 11.0–11.6] L/minute; *P* < 0.01) (Figures 3 and 4). Hourly oxygen consumption and total oxygen consumption were 4.8-fold and 4.8-fold higher in HFNO patients compared with ventilated patients.

### Subgroup analysis.

The findings of the preplanned subgroup analysis, including 206 patients (Supplemental Table 1), did not change the results. Actual oxygen consumption was 5.3-fold higher in HFNO patients compared with ventilated patients (median 15.5 [11.7–20.8] versus 2.9 [1.8–4.0] L/minute; mean difference = 13.8 [95% CI: 13.5–14.2] L/minute; *P* < 0.01), and hourly oxygen consumption and total oxygen consumption were 5.3–fold and 5.3–fold higher (Figure 3 and Supplemental Figure 2).

## DISCUSSION

The findings of this international study in critically ill patients with acute hypoxemic respiratory failure due to COVID–19 can be summarized as follows: 1) actual oxygen consumption with HFNO greatly exceeds actual oxygen consumption in patients receiving ventilation; consequently 2) hourly and total oxygen consumption is much higher in patients receiving HFNO, a difference that persisted even when excluding patients that switched from HFNO to mechanical ventilation within the first two full days of ICU care.

Our study has several strengths. First, it was an international study, including patients admitted to the ICUs of three academic hospitals in two European countries. This increases the external validity of our findings. Second, we captured oxygen supplementation data every 2 hours, providing us the opportunity to calculate various measures of oxygen consumption in a fairly precise way. Indeed, to our best knowledge, this is the first study that estimated actual, hourly, and daily data on oxygen consumption in a cohort of critically ill COVID–19 patients receiving either HFNO or ventilation. In addition, data were collected by trained data collectors, and the amount of missing data was low; in addition, we strictly followed the predefined analysis plan. Finally, only high-end ICU ventilators were used, for noninvasive and invasive ventilation, meaning we had no differences in bias flow, oxygen delivery, and leak compensation between these two forms of mechanical ventilation.

Although oxygen has been listed in 2017 in the WHO Essential Medicines Lists for both adults and children to treat hypoxemia,^14^ it was already a scarce resource in limited-resource settings before the ongoing pandemic. In the past 2 years, the incessant surges of patients in need of oxygen supplementation created local oxygen shortages in both resource-limited settings and high-income countries.^15^ For this reason, HFNO use was restricted in India during the second surge of the COVID–19 pandemic.^16^ Although information on the exact reasons for oxygen shortages at a local level remain scarce, there are obvious reasons for it. For instance, there have been reports of shortages due to problems with the delivery of oxygen to healthcare facilities, related to high costs or unpaid bills. Also, there have been reports on lack of oxygen cylinders, for example, because healthcare providers themselves started stockpiling and hoarding them.^7^ Another example is related to the poorly organized electricity arrangements, wherein electricity outages cannot be dealt with at a local level. This is especially problematic if oxygen supplementation depends on local production, at the hospital in a local plant or near the patient with bedside oxygen concentrators, which all need electricity.

One previous study reported data regarding oxygen consumption under experimental condition for noninvasive and invasive ventilation.^17^ The actual oxygen consumption in that study is comparable to our results; however, oxygen consumption during HFNO was not measured, and to our best knowledge there are no comparable studies in patients receiving HFNO. Differences between that study and our study in the amounts of oxygen consumed can be explained by the simplified formula to calculate the oxygen consumption we used, but maybe also the more precise description of oxygen consumption, including changes over hours in our study.

The findings of this study provide valuable input to the discussion on how to guarantee local oxygen supplementation. Hospitals can decide to install a liquid oxygen storage tank or a pressure swing adsorption plant. According to our results, the oxygen consumption per day is 850 L/hour * 24 hours ∼20,000 L during HFNO and 176 L/hour * 24 hours ∼4,200 L during noninvasive or invasive ventilation. Therefore, a full liquid oxygen tank of, for example, 10 K liters, which equals 8,500 K liters of gaseous oxygen, will be sufficient for ∼40 days of oxygen support for an ICU with 10 COVID–19 patients receiving exclusively HFNO, and ∼200 days of oxygen support for an ICU with 10 COVID–19 patients exclusively receiving noninvasive or invasive ventilation.^11^ A pressure swing adsorption plant could be more attractive, although it should be noted that output is affected by altitude.^18^ A plant that produces 1,000 L per minute is sufficient for oxygen support in ∼70 patients or more receiving HFNO, and more than 330 patients receiving noninvasive or invasive ventilation in case of uninterrupted production.

Departments may need to take care of local oxygen storages, or production. A full steel oxygen cylinder of, for example, 50 L, which equals up to almost 8,000 L of gaseous oxygen, will be sufficient for ∼9 hours of oxygen support for a COVID–19 patient receiving exclusively HFNO, and ∼44 hours of oxygen support for a COVID–19 patient receiving exclusively noninvasive or invasive ventilation. Here, an oxygen concentrator could be more attractive to use, but a bedside oxygen concentrator can produce only up to 15 L/minute. Of note, oxygen concentrators do not have batteries, meaning that electricity outages must be prevented, or covered by a local generator or other source. If not, enough filled oxygen cylinders should be present as a backup oxygen source.

Oxygen-sparing strategies include automated oxygen titration techniques,^19^^,^^20^ better bedside use of guidelines with strict cutoffs for SpO_2_ targets,^21^^,^^22^ and the use of prone positioning, in awake patients, that is, patients receiving HFNO^23^^–^^26^ or noninvasively ventilated ventilation,^27^ and invasively ventilated patients.^28^ The amount of oxygen spared with all these measures has yet to be studied and could be rather small, but any reduction in consumption, no matter how small, can help prevent a local “oxygen infarction” when applied on a larger scale. In an unfortunate situation of severe oxygen deficiency, it can of course be decided to stop applying HFNO as an intervention to avoid or postpone mechanical ventilation, so that only intubation would follow oxygen supplementation via low-flow systems in the treatment algorithm; if this is not necessary, the assessment of the infrastructural requirements regarding oxygen supply during HFNO remains of vital importance. Finally, it could be useful to identify patients who will fail HFNO, so that these patients can be switched to invasive ventilation at an earlier time point to spare oxygen.^29^^,^^30^

This study has limitations. First, we did not collect data of patients receiving lower flow oxygen support other than noninvasive or invasive ventilation, which could also substantially contribute to oxygen use in the ICU. Second, we may have underestimated oxygen consumption in ventilated patients in our study. Indeed, the simplified formula we used does not consider bias flow, which may be high in older ventilators, and leakages. Third, the study population only included patients with acute hypoxemic respiratory failure due to COVID–19, meaning that most patients had single-organ (i.e., respiratory) failure. Patients with respiratory failure due to other causes may respond less well to HFNO and may even need support with other levels of FiO_2_. Also, we restricted data collection to the first two full days of HFNO or ventilation, which usually lasts longer than our time frame. Fourth, our study was performed at a low altitude, and findings are not easily generalizable to hospitals at high altitude. Of note, we included only academic hospitals in high-income countries, where care for patients—particularly oxygen titrations—could be different from that in nonacademic hospitals in low-income countries (e.g., due to the lower number of healthcare providers at each bed). Fifth, we did not capture data about prone positioning, which prevents us from drawing conclusions about the effect of proning on oxygen consumption. Last, the retrospective nature of our study did not allow us to see how well HFNO was provided and how FiO_2_ was titrated. However, this can also be seen as a strength because it allowed us to calculate oxygen consumption in a realistic scenario.

## CONCLUSION

Oxygen consumption with HFNO greatly exceeds oxygen consumption with mechanical ventilation in the first days of ICU care for patients with acute hypoxemic respiratory failure due to COVID–19. This information may help hospital systems in planning the oxygen supplies, especially during high demand periods.

## Financial Disclosure

Amsterdam University Medical Centers, location Academic Medical Center; Netherlands Organization for Health Research and Development [ZorgOnderzoek Nederland/Medische Wetenschappen (ZonMw)].

## Supplemental Materials


Supplemental materials
