# Supplementary material for: Oxygen Consumption with High-Flow Nasal Oxygen versus Mechanical Ventilation— An International Multicenter Observational Study in COVID–19 Patients (PROXY–COVID)
Source: Am J Trop Med Hyg. 2023 Mar 27;108(5):1035–41. doi: 10.4269/ajtmh.22-0793 (PMC10160903; doi:10.4269/ajtmh.22-0793)
Supplement: Supplementary file 1 [file tpmd220793.SD1.pdf]

ONLINE SUPPLEMENT

Oxygen Consumption with High–flow Nasal Oxygen versus Mechanical Ventilation—an international multicenter observational study in COVID–19 patients (PROXY–COVID)

Michela Botta[1], Oriol Caritg[2], David M.P. van Meenen[1], Andres Pacheco[2], Anissa M. Tsonas[1], Willemijn E. Mooij[1], Alessia Burgener[1], Tosca Manrique Hehl[1], Gentle S. Shrestha[3], Janneke Horn[1,4], Pieter R. Tuinman[5], Frederique Paulus[1,6], Oriol Roca[7,8,9], Marcus J. Schultz[1,10,11]

Amsterdam University Medical Centers, Amsterdam, The Netherlands:
[1]Department of Intensive Care, location AMC
[5]Department of Intensive Care, location VUmc

Vall d'Hebron University Hospital, Barcelona, Spain
[2]Department of Intensive Care

Tribhuvan University Teaching Hospital, Maharajgunj, Kathmandu, Nepal
[3]Department of Critical Care Medicine

Amsterdam UMC Research Institute, Amsterdam, The Netherlands
[4]Amsterdam Neurosciences

Amsterdam University of Applied Sciences, Faculty of Health, Amsterdam, The Netherlands
[6]Urban Vitality, Centre of Expertise

Parc Taulí de Sabadell University Hospital, Barcelona, Spain
[7]Department of Intensive Care

Universitat Autònoma de Barcelona, Bellaterra, Spain
[8]Departament de Medicina

Instituto de Salud Carlos III, Madrid, Spain
[9]Ciber Enfermedades Respiratorias

Mahidol University, Bangkok, Thailand
[10]Mahidol–Oxford Tropical Medicine Research Unit (MORU)

University of Oxford, Oxford, United Kingdom
[11]Nuffield Department of Medicine

Table of Contents

eTable 1: Baseline Patient Characteristics (preplanned subgroup analysis) .... page 4

eFigure 1: Mode of oxygen supplementation over time .... page 5

eFigure 2: Cumulative frequency distribution of actual oxygen use, hourly oxygen use, and total oxygen use (preplanned subgroup analysis) .... page 6

eTable 1. Baseline Patient Characteristics (preplanned subgroup analysis)

	HFNO patients (N = 88)	ventilated patients (N = 118)	P
Age, years	60 [51, 70]	66 [59, 72]	<0.01
Male gender, no (%)	56 (65.1)	86 (72.9)	0.28
Body mass index, kg/m^2	29.3 [26.1, 31.7]	28.5 [24.9, 32.3]	0.27
SAPS II*, n/N	(78/88) 33 [26, 37]	(94/118) 50 [41, 64]	<0.01
Comorbidities, no (%)			
arterial hypertension	33 (37.5)	54 (45.8)	0.26
cardiovascular disease	16 (18.2)	27 (22.9)	0.49
diabetes mellitus	20 (22.7)	31 (26.3)	0.63
chronic kidney disease	12 (13.6)	12 (10.2)	0.51
pulmonary disease	9 (10.2)	19 (16.1)	0.30
malignancy	9 (10.2)	10 (8.5)	0.81
Home medication, no (%)			
systemic corticosteroids	10 (11.5)	7 (5.9)	0.20
ACE inhibitors	13 (14.9)	27 (22.9)	0.21
angiotensin II receptor blockers	14 (16.1)	14 (11.9)	0.42
beta blockers	16 (18.4)	24 (20.3)	0.86
insulin	8 (9.2)	17 (14.4)	0.29
oral antidiabetics	13 (14.9)	22 (18.6)	0.57
statins	23 (26.4)	34 (28.8)	0.75
calcium channel blockers	16 (18.4)	22 (18.6)	1.00
anticoagulation	8 (9.2)	14 (11.9)	0.65
ICU mortality, no (%)	12 (13.6)	50 (42.4)	<0.01

Data are medians (plus interquartiles) or number (with %). *SAPS II was not available for all patients. Abbreviations: HFNO = high flow nasal oxygen; NIV = noninvasive ventilation; SAPS = Simplified Acute Physiology Score; ICU = Intensive Care Unit.

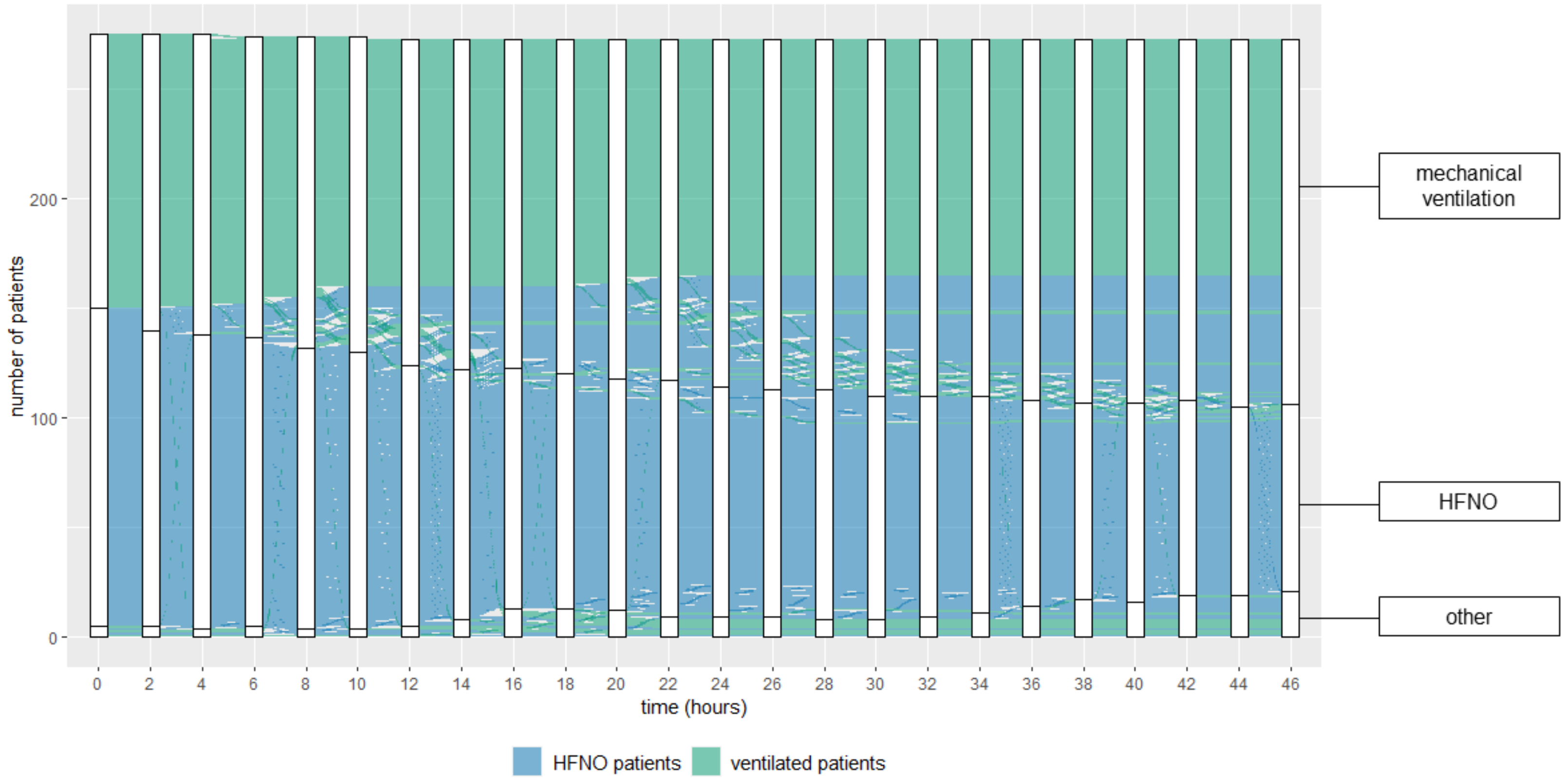

eFigure 1. Mode of oxygen supplementation over time
Abbreviations: NIV = noninvasive ventilation; HFNO = high flow nasal oxygen

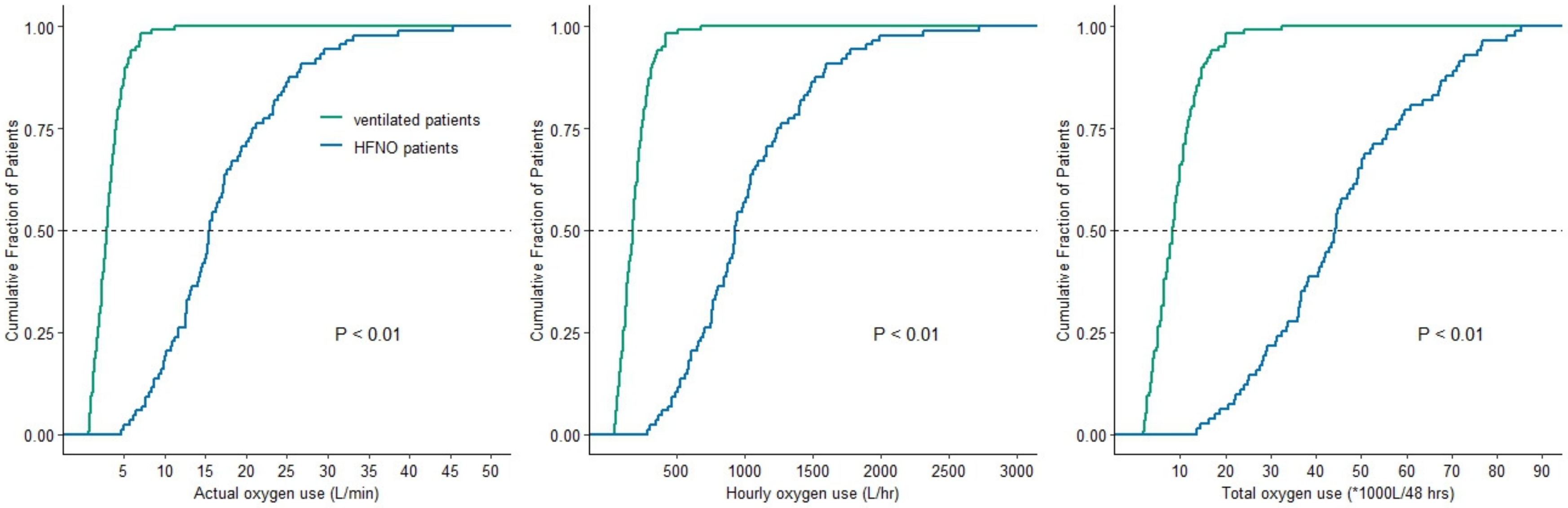

eFigure 2. Cumulative frequency distribution of actual oxygen use, hourly oxygen use, and total oxygen use (preplanned subgroup analysis)
P values refer to the Wilcoxon–Mann–Whitney test
